# Food Fortification: The Advantages, Disadvantages and Lessons from *Sight and Life* Programs

**DOI:** 10.3390/nu13041118

**Published:** 2021-03-29

**Authors:** Rebecca Olson, Breda Gavin-Smith, Chiara Ferraboschi, Klaus Kraemer

**Affiliations:** Sight and Life, P.O. Box 2116, 4002 Basel, Switzerland; breda.gavin-smith@sightandlife.org (B.G.-S.); chiara.ferraboschi@sightandlife.org (C.F.); klaus.kraemer@sightandlife.org (K.K.)

**Keywords:** fortification, micronutrients, micronutrient deficiencies, large-scale food fortification, biofortification, point-of-use fortification, micronutrient supplements, partnerships

## Abstract

Deficiencies in one or more micronutrients such as iron, zinc, and vitamin A are widespread in low- and middle-income countries and compromise the physical and cognitive capacity of millions of people. Food fortification is a cost-effective strategy with demonstrated health, economic and social benefits. Despite ongoing debates globally and in some countries regarding the performance and safety of food fortification, the practice offers significant benefits across each of the main vehicles for food fortification (large-scale food fortification, biofortification and point-of-use or home fortification) ranging from reducing the prevalence of nutritional deficiencies and economic benefits to societies and economies. Using *Sight and Life*’s global and national experiences in implementing food fortification efforts, we demonstrate how different programs in LMICs have successfully addressed challenges with food fortification and in doing so, find that these efforts are most successful when partnerships are formed that include the public and private sector as well as other parties that can provide support in key areas such as advocacy, management, capacity building, implementation and regulatory monitoring.

## 1. Introduction

Maternal and child undernutrition cause 45% of all deaths in children under five in low- and middle-income countries (LMICs) [1]. A particularly widespread problem in LMICs is hidden hunger, or a chronic lack of essential vitamins and minerals in the diet [2]. Deficiencies in one or more micronutrients such as iron, zinc, and vitamin A compromise the physical and cognitive capacity of millions of people. Overall, it has been estimated that micronutrient deficiencies account for about 7.3% of the global burden of disease, and iron and vitamin A deficiency rank among the 15 leading causes of the global disease burden [3], contributing to the deaths of over one million children annually [1]. The World Health Organization (WHO) estimates that 42 percent of children less than 5 years of age and 40 percent of pregnant women worldwide are anemic [4]; pregnant women with severe anemia are twice as likely to die during or shortly after pregnancy than non-anemic women, and for their children, micronutrient deficiencies in utero can lead to low birth weight and brain and spinal defects [5].

There have been a few attempts to quantify micronutrient malnutrition globally, but this has been hampered by insufficient intake and status data at the population level. While Schmidhuber et al. (2018) reported a generally increased supply of micronutrients between 1980 and 2013, Ruel-Bergeron et al. (2015) observed only a slight improvement of the Hidden Hunger Index (HHI) [6] from 1995 to 2011, globally (−6.7 net change on the HHI), and in Africa—particularly Sub-Saharan Africa—a deterioration in hidden hunger (+1.9 on the HHI) over the same period [7,8].

Micronutrient deficiencies are due primarily to inadequate intake of nutrient dense foods and nutrient losses due to poor diets, infections and blood loss during menstruation (women of reproductive age). Metabolic requirements for micronutrients are especially high during early development, pregnancy and lactation. The World Health Organization (WHO) and the United Nations Food and Agriculture Organization (FAO) have adopted four main strategies for improving dietary intake: food fortification, micronutrient supplementation, nutrition education, and disease control measures. The fortification of staple foods is one strategy that has a proven history in improving dietary diversity and effectively decreasing micronutrient deficiencies.

This review systematically synthesizes the available evidence on the advantages and disadvantages of food fortification vehicles (industrial fortification, biofortification and point-of-use or home fortification) and provides an overview of three dimensions by which fortification impacts individuals and societies—social, health and economic. It also provides examples and lessons learned from *Sight and Life* programs and projects that have worked to address barriers and enablers to food fortification across multiple dimensions.

## 2. What Is Food Fortification?

Food fortification is defined as the practice of adding vitamins and minerals to commonly consumed foods during processing to increase their nutritional value. It is a proven, safe and cost-effective strategy for improving diets and for the prevention and control of micronutrient deficiencies. In 2008 and 2012, the Copenhagen Consensus ranked food fortification as one of the most cost-effective development priorities [9,10]. While mandatory food fortification has been used as a strategy to prevent micronutrient deficiencies in high-income countries (HIC)—dating as far back as the 1920s in Europe and North America when the first salt was iodized—it is still less common in LMICs where food systems are not delivering nutritionally adequate diets due the production and consumption of just a few major starchy food crops (maize, rice, wheat) with low micronutrient content and/or bioavailability (phytate). In the past two decades, food fortification has become increasingly popular in LMICs for several reasons, including rapid urbanization and increasing household purchasing power, leading to a greater proportion of the population relying on processed foods [11,12].

There is strong evidence that food fortification in HICs is effective in addressing micronutrient deficiencies. In LMICs, the evidence is still being established, but research, including a recent systematic review and meta-analysis of large-scale food fortification (LSFF) programs, confirmed the impact of fortification on nutritional outcomes including reductions in vitamin A deficiency, iodine deficiency, anemia, and iron deficiency among women and children; declines in goiter and neural tube defects (NTDs) among children; and improved serum folate among women of reproductive age [13]. However, certain fortification approaches, such as iron fortification of staple foods like flour, provided mixed results. For example, Field et al. (2020) found that fortifying wheat flour with iron had little or no effect on anemia [14] and had little to no impact on iron deficiency compared to unfortified wheat flour; and that the addition of other micronutrients also had little impact on iron deficiency and hemoglobin concentrations compared to wheat flour alone [15]. Similarly, Pachon et al. (2015) found limited evidence to support the reduction of anemia with large-scale flour fortification [16]. In contrast, Barkley et al. (2015) found that among countries with national flour programs, there was a 2.4% reduction in the (odds) of anemia for non-pregnant women in each year of fortification in comparison to the previous year [17].

The type of fortification that will be most appropriate and effective in a given country depends on several factors including: the prevalence of certain micronutrient deficiencies, the population(s) most affected, dietary compositions, available infrastructure, capacities for food processing and production systems, as well as national regulation and governmental leadership. The main fortification vehicles are as follows. 

### 2.1. Large-Scale Food Fortification

Industrial or large-scale food fortification (LSFF) is the addition of micronutrients during processing to commonly consumed foods such as salt, flours, oil, sugar and condiments. LSFF programs can be categorized as either mandatory—meaning they are initiated and regulated by the government—or voluntary where food processors add nutrients to their foods on their own volition but is still governed by regulatory limits. Mandatory fortification programs are increasingly common, especially when it comes to fortified flour and iodized salt. Salt iodization is perhaps the most common form and between 1990 and 2008, the number of households globally consuming iodized salt rose from 20 percent to 70 percent [18]. Currently, over 130 countries have mandated iodized salt [19].

Mandatory wheat flour fortification was first introduced in 1942 and currently 85 countries have since mandated its use. In North and South America, addition of folic acid to wheat flour is mandatory to lower the risk of birth defects. Edible oils are an increasingly common vehicle for fortification, and thus far 27 countries have mandated oil fortification with Vitamin A; and 14 countries have mandated milk fortification, 11 countries fortify milk with both Vitamin A and D, one country (Costa Rica) is additionally fortifying with iron and folic acid, and two countries (China and Canada) are adding calcium, in addition to Vitamin A and D [20]. 

Sugar fortification with vitamin A began in the 1970s in Latin America and was first implemented in Guatemala in 1975 becoming a model for other countries because it resulted in a near tripling of vitamin A intake and a decrease in vitamin A deficiency from 22 to 5% in only one year [21]. In Africa, there are mandatory sugar fortification programs in Malawi, Mozambique, Nigeria, Rwanda, Zambia and Zimbabwe.

Currently, over 140 countries globally have guidance or regulations in place for fortification programs, the majority of which are mandatory, and almost 140 countries are implementing national salt iodization programs of which 102 are mandatory, 85 countries mandate at least one kind of cereal grain (maize, rice or wheat) be fortified with iron and folic acid, and over 40 countries mandate the fortification of edible oils, margarine and/or sugar with vitamin A and/or vitamin D [22].

Food fortification can also be achieved by harnessing the expertise of the private sector to produce and distribute fortified foods. Voluntary fortification is the process by which a food manufacturer chooses to add one or more micronutrients to processed foods in compliance with government regulations and standards. An example of this is Olam in Ghana who is fortifying long-grain rice with micronutrients including iron, zinc, and B-complex vitamins, providing more than 15% of the minimum RDA (recommended dietary allowance) per serving [23]. In India and Kenya, voluntary fortification initiative were drivers of more exhaustive legislation and a strong enabling environment [24].

### 2.2. Biofortification

In contrast to LSFF where nutrients are added during post-harvest processing, biofortification is the process by which food crops are grown to improve their nutritional value. Biofortification projects mainly concentrate on boosting iron, zinc and provitamin A carotenoid in different food crops through plant breeding or agronomically (mineral fertilizer); some projects have also biofortified with amino acids and protein. Examples of biofortification projects include iron biofortification of rice, beans, maize and sweet potato; zinc biofortification of wheat, rice, beans, sweet potato and corn; and Vitamin A biofortification of sweet potatoes, corn and cassava. 

Research on biofortification has identified the advantages of this approach, specifically it targets poor families living in remote rural areas with no or limited access to industrially fortified foods. These families often rely on subsistence farming and can grow, consume and sell their own fortified crops. Additionally, when targeted correctly, biofortification can enable food systems to deliver more nutritious foods cost-effectively [25]. In northern Mozambique, a 2019 study [26] found that the introduction of orange sweet potatoes (OSP) to farmers increased vitamin A intake among women of reproductive age and children, and improved vitamin A intake (with long-term impacts on vitamin A intake). Nutrient poor diets based on staple crops (tubers and cereals) typically lead to multiple micronutrient deficiencies, but biofortification can increase a crop’s nutritional value using traditional breeding and agronomic biofortification techniques. However, biofortification via genetic engineering—which allows for simultaneous introduction of multiple micronutrients in a single crop—could support increased levels of multiple micronutrients in a single food crop and high-level accumulation of micronutrients [27].

### 2.3. Point-of-Use or Home Fortification

Point-of-use fortification is the addition of vitamins and minerals to food that has been cooked and is ready to be eaten. Formerly known as “home fortification”, the WHO adopted the term “point-of-use” in 2012 to reflect the many settings where this type of intervention can take place such as in schools and refugee camps; and in 2016, recommended point-of-use fortification of complementary foods with micronutrient powders (MNPs) as a key intervention for improving micronutrient intake (to improve iron status and reduce anemia in particular) in children aged 6–24 months [28].

MNPs are single-dose packets containing multiple vitamins and minerals in powder form that can be sprinkled onto food without affecting the taste or color [29]. The original purpose of “Sprinkles” (original branding) was to provide iron and other nutrients for treating anemia and iron deficiency but the formulations have since changes to address the multiple nutrient needs of children under five [30]. Currently, most countries use a 15 micronutrients MNP formulation designed to provide one Recommended Nutrient Intake (RNI) of each micronutrient per dose for children 6–59 months old [31]. Each sachet costs approximately USD 0.2, [31] and is recommended in settings where children have low dietary diversity, locally available foods have low nutritional value, or when a child has infectious diseases (malaria, diarrhea, worms) [30]. 

A recent Cochrane review has established that MNPs are effective in reducing anemia and iron deficiency for children 6–24 months [30]. Home fortification with MNP reduced anemia by 31% and iron deficiency by 51% in infants and young children when compared with no intervention or placebo but did not find an effect on growth [32]. Research by the World Food Programme, using Save the Children’s Cost of Diet tool, also found that the addition of two sachets of MNPs to the diet in Indonesia could reduce the amount that families would need to spend to obtain an adequately nutritious diet by 20% [33].

Malnutrition in all its forms affects millions of women worldwide, but women in LMICs are particularly vulnerable to deficiencies in essential vitamins and minerals such as iron, folic acid, zinc and iodine due to poor quality diets. These nutritional deficiencies not only affect their health but also that of their children. Latest estimates reveal that 154 million women of reproductive age are too thin and 520 million women are anemic, two conditions that are linked to the birth of 20 million low birth weight (LBW) babies annually [34]. Maternal micronutrient supplements (MMS) are a safe and cost-effective solution to improve the nutritional status of pregnant women in LMICs [35]. Studies show that MMS reduces LBW up to 13% and SGA by 9% [36].

## 3. Advantages and Disadvantages of Food Fortification

### 3.1. Health

**Figure 1:**
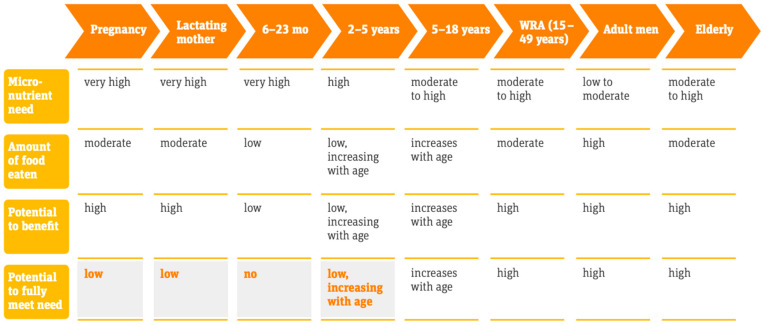
Potential benefits of food fortification across the life cycle. Source: Irizarry, L, Prost, MA, Murillo, D, Lopez de Romaña Daniel et al. 2017. Scaling Up Rice Fortification in Latin America and the Caribbean. World Food Programme and *Sight and Life*: 2017. WRA = Women of Reproductive Age.

According to WHO mortality data, around 0.8 million deaths (1.5% of the total) can be attributed to iron deficiency each year, and a similar number to vitamin A deficiency [37], and contributes to a significant number of lives lost [38]. A significant body of literature shows that LSFF can have public health impacts in HICs as well as in LMICs. A recent analysis of 50 studies in LMICs has shown that fortification with iodine, folic acid, vitamin A and iron have led to dramatic reductions in serious disease [36]. A 2019 review attempted to estimate the real-world impact of industrial food fortification on health and nutrition outcomes in LMICs, and found that it had a positive impact on some health outcomes, including goiter, anemia, and NTD prevalence [36]. Fortification programs implemented population-wide were associated with a 34% reduction in anemia from improved iron stores, with greater benefits realized by those most at risk of deficiency; 74% reduction in the odds of goiter; and a 41% reduction in the odds of NTDs [14]. Figure 1 shows the potential benefits of food fortification across the life cycle. 

Numerous country-level studies on the impact of food fortification on micronutrient status have shown very positive results. For example, in Indonesia, a study conducted in two districts of West Java from 2011 to 2012 assessed the effects of large-scale fortification on the vitamin A status of women and children and found that fortified oil increased vitamin A intake close to the recommended nutrient intakes, contributing on average 26% of daily need for children aged 12 to 23 months, 38–40% among older children, and 29–35% for women [39]. Not only did the vitamin A status of all beneficiaries improve and vitamin A deficiency dropped significantly, but so the vitamin A content of breast milk of lactating mothers also increased. In Costa Rica, an evaluation of the impact of iron fortification on anemia prevalence found a significant decrease at the national level in the prevalence of anemia among children aged 1–7 years and women of reproductive age. Anemia was reduced from 19 to 4% in children and from 18 to 10% in women; and in children, iron deficiency was also reduced from 27 to 7% [40].

Despite the enormous benefits of food fortification strategies on nutritional status, some studies have identified opposite results in terms of no impact of food fortification programs and of guaranteeing safe upper limits. For example, a study conducted among Brazilian children under the age of six found no effect of iron-fortified flour on anemia prevalence. The study consisted of four population-based surveys conducted over a four-year period, and it measured dietary intake and hemoglobin levels. The findings showed an unexpected increase in anemia among children. Despite the average intake of fortified flour detected by the study amounting to 100 g per day, the poor diets quality of children with low bioavailability of iron compromised the benefits of fortified flour [41].

Furthermore, a systematic review performed in both HICs and LIMCs did not find any significant association between the effect of multiple micronutrient fortification on child growth outcomes such height/length-for-age z-score (HAZ/LAZ)) (MD 0.09, 95% CI 0.01 to 0.18; 8 studies, 2889 participants; low-quality evidence) and zinc deficiency (RR 0.84, 95% CI 0.65 to 1.08; 5 studies, 1490 participants; low-quality evidence) [20]. Fortified complementary foods had a small effect on children’s anemia but had no impact on their growth and in some cases, they were associated with more diarrhea episodes [42].

There are also ongoing debates globally and in some countries regarding the performance and safety of fortification efforts (whether or not the consumption of micronutrient fortified foods may cause adverse health effects due to the accumulation of these nutrients in the human body). To ensure success in food fortification, the micronutrients’ Tolerable Upper Intake Level (UL) must be established. The WHO proposed a methodology for calculating and defining the safe upper limit in the Guidelines on Food Fortification with Micronutrients, which vary depending on the local context. However, challenges can arise in case of large consumption of the fortified food among the same population and utilization of multiple food vehicles [41]. In Guatemala it was shown that the consumption of wheat flour fortified with acid folic varied massively among people from different socioeconomic status (women from wealthy groups consumed 15 times more than women from no wealthy groups) rising concerns on the safe limits [43]. In Cameroon, the consumption of multiple Vitamin A fortified vehicles such as sugar or wheat flour with edible oil resulted in a possible excess of UL among children. This was the case in urban settings due to the high consumption of such foods [44].

While some evidence suggests that while multiple exposures of micronutrients including fortified foods for a long period of time may result in adverse effects, when properly regulated, fortification carries a minimal risk of toxicity [45]. For example, Vitamin A fortification safety is often cited as a concern in food fortification programs, but a recent review indicated that the risk of excessive vitamin A consumption from fortified foods in women and young children is likely negligible [46].

### 3.2. Economic

Food fortification is a cost-effective strategy to improve the nutrition status of populations [47,48,49], and it associated with high economic benefits [50,51,52,53]. Reviews such as the Copenhagen Consensus have consistently ranked micronutrients as the most cost-effective development intervention and provides significant returns for a low cost [11]. For example, iodizing salt can cost as little as USD 0.05 and wheat and maize fortified with iron and folic acid as little as USD 0.12 annually. Lifetime costs for these two fortified commodities are less than USD 15 per person and can provide a return on investment of more than USD 26 in increased productivity and health care savings; and every dollar spent on fortification results in USD 9 in benefits to the economy [10].

A feasibility and cost-effectiveness study in the Philippines using fortified powdered milk to increase micronutrient intake amongst children found it as a cost-effective tool for addressing iron deficiency [54]. The results indicated that for iron deficiency, food fortification was the most cost-effective method with a cost of USD 66 per disability adjusted life years (DALY), whereas supplementation and dietary diversification had estimated costs of USD 179 and 103 per DALY, respectively. In the case of biofortification, the estimated health benefit-to-cost ratio was USD 17 of benefits for every USD 1 invested [11].

Despite the enormous economic potential of food fortification, several barriers are not conducive to creating an enabling environment for global food fortification scale-up. These constraints include low private-public partnership and lack of national regulations on food fortification. A recent systematic review addressed the challenges of LSFF programs in LMICs, in addition to the effectiveness of food fortification programs. The authors identified engagement with Small and Medium Enterprise as a bottleneck to expanding food fortification programs, as well as their low technological and economic capacity; lack of regulations and food laws were also cited as barriers [47]. For example, despite mandatory salt iodization in the Philippines, more than two-thirds of the salt is imported and not fortified due to low production capacity. Furthermore, the national production of salt is carried out by producers without a license, thereby avoiding any official controls; and the absence of high-quality laboratories prevent control and monitoring to assess the quality of fortified food [55]. Luthringer et al. provide a comprehensive analysis of regulatory monitoring on fortified foods. The paper emphasized conflicts and best practices for governments and the food industry in LMICs. Key aspects of successful fortification strategies include partnership between Governments and food industries and developing the economic and human capacities of small industries [56].

### 3.3. Social

As outlined in the 1996 declaration of the World Food Summit: “Everyone to have access to safe and nutritious food, consistent with the right to adequate food and the fundamental right of everyone to be free from hunger” [57]. The right to food is also recognized in the 1948 Universal Declaration of Human Rights and is enshrined in the 1966 International Covenant on Economic, Social and Cultural Rights. As such, combatting hunger and malnutrition in many countries is more than a moral duty but is a legally binding part of national law under human rights obligation. Unfortunately, guaranteeing the right to food and nutrition is a complex and often overwhelming challenge in many LMICs. Particularly in the context of the COVID-19 pandemic in which mandatory lockdowns and economic slowdowns are leading to increases in food insecurity and malnutrition. Therefore, food fortification can be a crucial intervention to decrease the risk of malnutrition—before, during, and after pandemic.

The Sustainable Development Goals (SDGs) and the 2030 Agenda for Sustainable Development (2030 Agenda) aim to achieve a world free of hunger and malnutrition in all its forms, and to use data and global partnerships to realize the goals. In fact, SDG17 encourages “the global partnership for sustainable development, complemented by the use of multi-stakeholder partnerships” as a means of implementation of the 2030 Agenda [58]. Food fortification is a key element to strengthen the public and private partnership with beneficial multi-sectoral impacts on society. 

While private sector partners from the food industry are the main actors within food fortification programs, governments and civil society also have a role to play. Civil society in particular can help improve accountability and commitment to standards, etc. Public-private partnerships (PPPs) are important for food fortification programs as most/all programs have some link between the public and private sector, as well as engagement with consumers, civil society, donors, etc. 

Finally, food fortification, when combined with social safety net programs (SSNP), such as school feeding programs, distributions to the poor or to vulnerable groups, food for work programs, and food aid during emergency situations, have been effective tools to deliver fortified food to vulnerable people but also disseminate information on diets [59]. In Gujarat, India, the government used three social safety net programs—Gujarat’s Public Distribution System (PDS), Integrated Child Development Scheme (ICDS), and Mid-Day Meal (MDM) Programme—as platforms for introducing wheat flour fortification among beneficiaries. In doing so, they found that by substituting wheat grain for fortified wheat flour dramatically increased the intake of micronutrients among its SSNP beneficiaries, and that it was a very cost-effective approach [60].

A challenge for school feeding and other SSNP programs is their long-term effectiveness. In South Africa, in nearly four years of implementation of a Vitamin A fortified biscuits in school program, vitamin A deficiency has not been eradicated despite the biscuits provided 50% of daily RDA for carotene and 10% of the daily RDA for vitamin A [61]. Another challenge emerged during the recent lock-downs due to the COVID pandemics when SMEs faced increased difficulties to access to the vitamins and mineral premix for the fortification procedures, particularly from the international premix supply [62].

## 4. Lessons from *Sight and Life* Projects and Partnerships

Global and national experiences show that food fortification accomplishments are most promising when partnerships are formed not only between the public and private sectors, but also with parties/organizations that can contribute in the following critical areas: advocacy, management, capacity building, implementation and regulatory monitoring. *Sight and Life* is a global humanitarian organization that works to end malnutrition in all its forms. The projects are grounded in evidence and build strategic and long-lasting partnerships to implement sustainable solutions to improve the lives of those in most need, and include several projects focused on food fortification. *Sight and Life* works as a catalyst in bringing key stakeholders together to address multidisciplinary challenges related to food fortification strategies. The following section will highlight some of key lessons learned from *Sight and Life* projects and its partners.

### 4.1. India—Mandatory Rice Fortification

Micronutrient deficiencies are common among women and children in the Indian state of Andhra Pradesh. In recognition of this challenge, in early 2018, the Prime Minister’s office launched the *National Nutrition Mission* where staple food fortification as a cost-effective approach to control vitamin and mineral deficiencies. Among the various staples available in Andhra Pradesh, rice is the most effective vehicle to reach the poorest and only one of two staples, which when fortified well, can carry a range of minerals and vitamins. It is delivered to the most nutritionally vulnerable population through the government’s three main food supplementation programs: (1) Mid-day Meal scheme (MDM), (2) Integrated Child Development Scheme (ICDS), and (3) Public Distribution System (PDS).

The Food Safety and Standards Authority of India (FSSAI) developed specifications for fortified rice after expert consultations which included strong clinical evidence from leading academic institutions in India. Since then, rice fortification has gained momentum and currently fifteen states have drawn up plans to start implementing it through MDM, ICDS and PDS. To strengthen these programs, *Sight and Life*, in collaboration with Tata Trust and the local Government, has instituted a promising, cost-effective blending process known as continuous blending. This is the first time that this type of approach has been implement in India for fortify rice under large-scale government programs.

### 4.2. Rwanda—Example of Industrial Fortification

In October 2019, the Government of Rwanda, through its Ministry of Health started a food fortification program of five staple foods, namely maize and wheat flour (fortified with vitamin A, iron, zinc, folic acid, niacin, vitamin B1 and vitamin B12), edible oils (fortified with vitamin A), sugar (fortified with vitamin A) and salt (fortified with iodine). Together with partners like *Sight and Life*, Rwanda has developed mandatory food fortification standards and have supported the process of passing new food fortification regulations making it mandatory for all locally and imported food products among the five food staples. Currently, some food processors like African Improved Foods, MINIMEX, and SOSOMA have introduced in the market fortified flours and some imported edible oils from neighboring countries are also fortified.

In 2019, Rwanda established their own Food and Drug Administration (FDA) and in collaboration with *Sight and Life* has established a food fortification platform where all stakeholders meet to discuss matters related to the implementation of the food fortification regulation. For example, through the Rwanda National Fortification Alliance, members identified VAT taxation as one of their biggest challenges, and key stakeholder groups such as the private sector, CSOs and NGOs supported discussions with the Ministry of Finance to reduce or wave VAT taxes on fortified flour as a key fortification incentive.

### 4.3. Ghana—Voluntary Fortification Example

In Ghana, micronutrient deficiencies among women and child remain high—a recent micronutrient survey conducted by the Ghana Health Service (GHS) revealed deficiencies in key micronutrients including vitamin A, iron and folate, particularly in pregnant women including that approximately 50 and 66% of non-pregnant and pregnant women and pre-school age children, respectively, suffer from anemia, and 30% of pre-school children suffer from iron and vitamin A deficiency [63].

In 2013, a partnership between *Sight and Life*, DSM, the German Federal Ministry for Economic Cooperation and Development, the Children’s Investment Fund Foundation (CIFF), Bill and Melinda Gates Foundation (BMGF), Association of Ghanaian Industries (AGI) and Ghana Standards Authority (GSA) was launched.

The program is a demand driven approach to addressing micronutrient malnutrition among women of reproductive age by creating a distinctive front-of-package seal that guarantees nutrition quality, while easily identifying fortified food products that provide a good source of 18 vitamins and minerals designed for women of reproductive age. The OBAASIMA Seal does not provide clear assurance to consumers of a high quality, safe, and nutritious food that adheres to the minimum fortification standards, but it also serves as a good model for engaging the private sector in LMICs to alleviate micronutrient deficiencies.

Moreover, the OBAASIMA project was used as case study to advocate for the importance of suitable nutrient profiling methods in LMICs. *Sight and Life* in collaboration with Professor Adam Drewnoski recently published a study calling for new nutrient profiling models appropriate for LMIC nutritional needs. The study showed that current nutrient profiling methods adopted in HIC fail to capture benefit of nutrient dense food such as fortified foods that meet the nutritional needs of people in the Global South. Guidelines for developing a Nutrient Profiling system in LMICs were [64].

## 5. Conclusions

Food fortification is a cost-effective intervention with the potential to address malnutrition globally. Studies on the fortification of foods have shown positive results not only in the control and prevention of micronutrient deficiencies among vulnerable populations, especially women and children, but also along social, economic and environmental dimensions. *Sight and Life* projects offer successful examples to address micronutrient deficiencies through fortification from several LMICs and emphasize the importance of multidimensional partnerships in addressing the many challenges of food fortification strategies. This paper aimed to demonstrate advantages and disadvantages of food fortification strategies, as well as to provide some key examples of ways in which different programs in LMICs have systematically addressed the issues identified, demonstrating that there is no one-size-fits-all solution.
